# The National Institute of Neurological Disorders and Stroke (NINDS) Epilepsy Therapy Screening Program (ETSP)

**DOI:** 10.1007/s11064-017-2275-z

**Published:** 2017-05-02

**Authors:** John H. Kehne, Brian D. Klein, Shamsi Raeissi, Shalini Sharma

**Affiliations:** National Institutes of Health/National Institute of Neurological Disorders and Stroke, Rockville, MD 20852 USA

**Keywords:** Epilepsy, Preclinical screening, Seizure models, Epileptogenesis, Disease modification, PANAChE, Epilepsy Therapy Screening Program (ETSP), Pharmacoresistance

## Abstract

For over 40 years, the National Institute of Neurological Disorders and Stroke/National Institutes of Health-funded Anticonvulsant Screening Program has provided a preclinical screening service for participants world-wide that helped identify/characterize new antiseizure compounds, a number of which advanced to the market for the treatment of epilepsy. The newly-renamed Epilepsy Therapy Screening Program (ETSP) has a refocused mission to identify novel agents which will help address the considerable remaining unmet medical needs in epilepsy. These include identifying antiseizure agents for treatment-resistant epilepsy, as well as anti-epileptogenic agents that will prevent the development of epilepsy or disease-modifying agents that will ameliorate or even cure established epilepsy and its comorbidities. This manuscript provides an overview of the ETSP’s efforts aimed at identifying the next generation of therapeutic agents to further reduce the suffering from and burden of epilepsy.

## Introduction

**Fig. 1:**
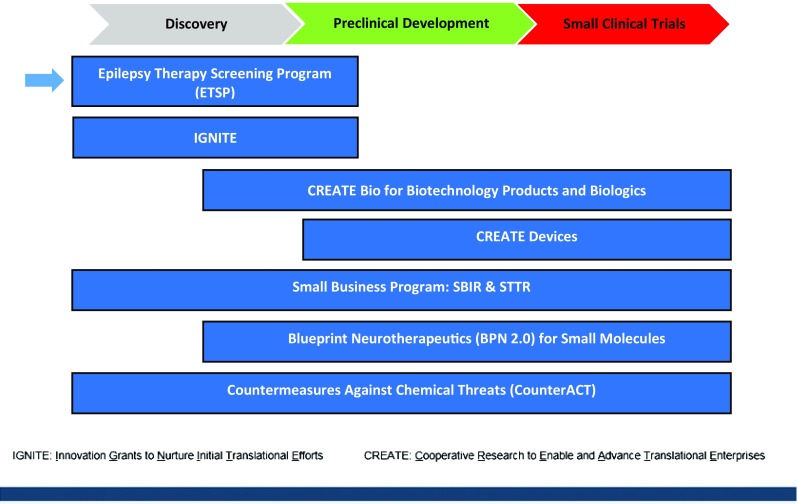
The Epilepsy Therapy Screening Program (ETSP) is part of the NINDS/NIH Division of Translational Research

The Epilepsy Therapy Screening Program (ETSP) is a National Institute of Neurological Disorders and Stroke (NINDS)/ National Institutes of Health (NIH)-funded, preclinical screening program with a mission to facilitate the discovery of new therapeutic agents addressing unmet medical needs in epilepsy. The ETSP is part of the NINDS Division of Translational Research (DTR) (Fig. 1) which has a mission to facilitate the discovery and development of new therapeutic interventions for neurological disorders. ETSP is the new name (implemented in 2016) for the longstanding program known world-wide to the epilepsy community as the Anticonvulsant Screening Program (ASP). The renaming of the program represents the refocusing and expansion of goals to encompass drug-refractory epilepsy, epileptogenesis and disease modification of epilepsy and its comorbidities. The purpose of this manuscript is to describe the current status of the ETSP, with reference to how the program has been modified to best achieve these challenging goals.

The ETSP is built on a strong foundation, the ASP, established in 1975 when considerable need existed for improved antiseizure drugs with fewer side effects and other liabilities associated with existing agents. The ASP was originally part of a larger NIH-funded Epilepsy Drug Development Program, which pursued both discovery and development of new agents, and the ETSP is the sole remaining component of that program today. For an in-depth historical perspective on the ASP/Anticonvulsant Drug Development Program the reader is referred to another manuscript in this special issue authored by Roger Porter and Harvey Kupferberg.

For over 40 years, the ASP has been run as a 5 year contract that is managed by the NINDS. The ASP has provided testing in a battery of rodent antiseizure tests at no cost to participants from the United States and other countries around the world. Since its inception, the contract has been continuously awarded to the University of Utah in Salt Lake City with faculty from the Department of Pharmacology and Toxicology (in the College of Pharmacy) serving as principal investigators. In 2001, H. Steve White, Ph.D., the honoree of this Special Issue of Neurochemical Research, assumed the principal investigator role which he held until 2016, when he became Chair of the Department of Pharmacy at the University of Washington in Seattle. During his longstanding involvement with the ASP, especially during his tenure as principal investigator, Dr. White was very instrumental in helping the NINDS achieve its ASP mission. Dr. White’s colleague and co-investigator, Karen Wilcox, Ph.D., assumed the role of principal investigator upon his departure and continues in that role for the new contract which was awarded to the University of Utah in September 2016 after an open competition. Dr. White continues to contribute to the program through a subcontract awarded to the University of Washington by the prime contractor.

**Fig. 2:**
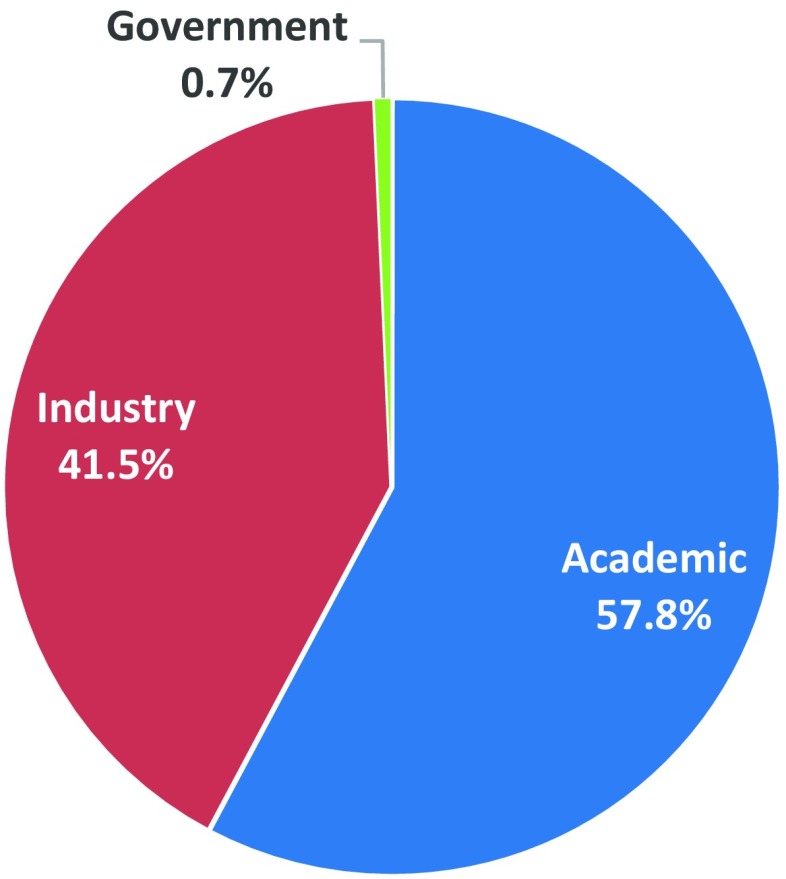
The Anticonvulsant Screening Program (ASP) has tested 32,000 compounds from 38 countries, representing participants from academia, industry, and government sectors

**Fig. 3:**
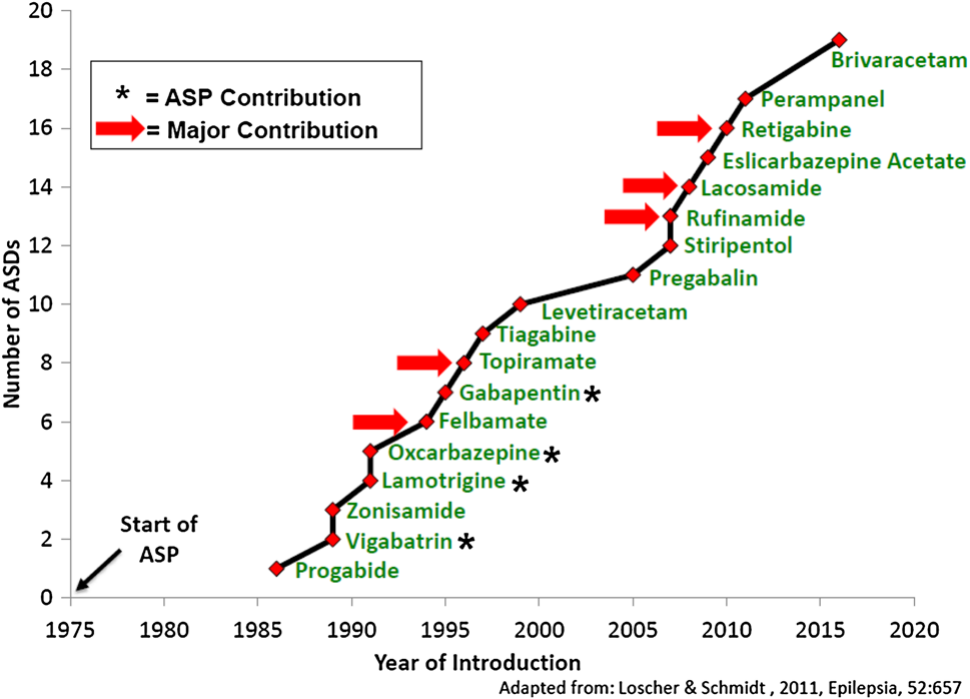
The Anticonvulsant Screening Program (ASP) contributed to the worldwide registration of antiseizure drugs (ASDs) for use in human epilepsy

Over the course of its history, the screening program has tested over 32,000 compounds submitted from more than 600 participants. The program has been truly international in scope, with 38 countries represented. The program participants have come from academia (~60%), or industry (~40%; pharma companies, usually small biotech companies, as well as large pharma companies), and there are a small percentage of participants from other government agencies (Fig. 2). The success of the program is highlighted by ASP’s contribution to the identification and/or characterization of nine drugs for the treatment of human epilepsy that have come to the market since 1990 (Fig. 3).

As has been noted elsewhere [1, 2], the success of the epilepsy drug discovery effort has been largely attributable to the predictive validity of the maximal electroshock seizure test in animals. This test, routinely performed in rodents and supplemented by several other acute chemically (e.g., subcutaneous metrazol) and electrically (e.g., 6 Hz stimulation) induced seizure models can effectively identify the majority of agents currently marketed for the treatment of epilepsy. While these acute tests have been effective in advancing the field, there remain unmet needs in epilepsy which will likely require new models, approaches, and the utilization of advanced technologies.

### Epilepsy Therapy Screening Program

The screening program is routinely reviewed by an external panel of experts which then make recommendations to NINDS regarding future directions. Most recently, Working Groups commissioned by the National Advisory Neurological Disorders and Stroke (NANDS) Council reviewed the ASP. The Working Groups were comprised of a distinguished panel of experts from academia, industry as well as other patient advocacy programs and non-profit organizations. The members of the most recent (2015) ASP Working Group were Robert Pacifici, Ph.D. (CHDI Foundation), Ray Dingledine, Ph.D. (Emory University, GA), Amy Brooks-Kayal, M.D. (Children’s Hospital of Colorado, CO), Henrik Klitgaard, Ph.D. (UCB Pharmaceuticals, Inc., Belgium), Elizabeth Kovar, Ph.D. (Univ. of Chicago, IL), Roy Twyman, M.D. (Johnson & Johnson, NJ), and Michelle Welborn, PharmD, ICE Epilepsy Alliance). The outcomes of the last two Working Group reviews are summarized in reports released in February 2012 and May 2015 (https://www.ninds.nih.gov/Current-Research/Focus-Research/Focus-Epilepsy/ETSP). From a scientific perspective, the Working Groups generally recommended that the program refocus and intensify its efforts on identifying compounds that will address the major areas of unmet medical need in epilepsy. These included identifying drugs for treating pharmacoresistant epilepsy (i.e., the 30% of persons with epilepsy not adequately treated with existing antiseizure drugs) as well as expanding into areas of disease prevention and modification, areas for which there are currently no FDA-approved drugs. Related to this, it was suggested that the program name be changed to reflect its expanded mission beyond finding new anticonvulsants, and to this end, the name “Epilepsy Therapy Screening Program” (ETSP) was formally adopted in 2016.

Another major recommendation was to establish an External Consultant Board (ECB) the members of which would provide ongoing individual feedback to the program. The ETSP ECB was established in May 2015 and is currently comprised of the following distinguished consultants: Wolfgang Löscher, Ph.D. (University of Hannover, Germany); Amy Brooks-Kayal, M.D. (Children’s Hospital of Colorado, CO); Henrik Klitgaard, Ph.D. (UCB Pharmaceuticals, Inc., Belgium); and Stephen Perrin, Ph.D. (ALS Therapy Development Institute, MA). This group formally interacts with the ETSP on a quarterly basis, with one of those meetings being a face-to-face, full day meeting at the NINDS site in Bethesda, MD. Importantly, the first part of an ECB meeting includes participation of the senior scientific staff from the prime contractor, thereby providing an opportunity for direct feedback on topics such as current test methodologies as well as ideas and proposals for new testing assays. In addition, the ETSP staff meet directly with the contractor senior scientific staff on a regular basis through web meetings and a yearly site visit to ensure optimal communication.

As noted above, the ETSP is a part of the NINDS Division of Translational Research, as will be discussed at greater length later in this manuscript. In recent years, in response to Working Group feedback, the ETSP has become more integrated with other programs within NINDS, including the Division of Neuroscience Research and the Division of Clinical Research. This internal multidisciplinary team is able to discuss information which may cross the boundaries between epilepsy and other neurological disease areas. Such interactions, combined with outreach through participation in external scientific meetings and close communication with the prime contractor, are intended to maximize the success of the ETSP in pursuit of its mission.

The following sections will focus on current scientific and operational aspects of the program, with occasional reference as to how the program has been modified in response to the Working Group recommendations and ongoing feedback from the ECB and other internal and external parties.

## Processes for Compound Selection, Submission, and Data Reporting

After a potential participant contacts the ETSP staff, an initial web meeting is set up to provide an opportunity for the investigator(s) to learn about the program and to convey their reasons for submitting compounds. Following mutual agreement to move forward, both the participant and NINDS sign a Participant Agreement form which outlines conditions for confidentiality, intellectual property protection, the compound submission process, and other considerations, and thereafter an in-depth discussion occurs whereby compound structures are revealed to NINDS ETSP staff, along with any relevant additional biological/chemical information. Each compound is given individualized assessment and examined carefully for inclusion for testing in the program. If accepted, the compound is registered through an internal process and information on identity/purity reviewed by the NINDS ETSP staff. In this context compounds submitted are predominantly small molecules but large biologics may be tested as well. A requisite for testing any investigational compound is that adequate documentation is provided for proof of identity (e.g., mass spec, NMR data) and purity (HPLC, elemental analysis data). The acceptable purity for a compound is ≥95%. If approved, the participant ships the compound to a designated compound management site where it is relabeled with a coded number (source/identity known only to NINDS ETSP staff), and shipped to the contract site for blinded evaluation. This latter “blinding” step is an important step as it prevents a potential bias afforded by staff at the site from knowing the structure/source of a test compound. When the compound is received at the contract site, an NINDS ETSP project manager develops a study design based on information provided by the participant, and places orders for specific tests to be performed at the contract site. When the in vivo or in vitro data are generated, they are reviewed by the ETSP project manager and communicated to the participant. Based on the test results, testing is either discontinued or next steps are planned for testing at the contract site. Interim and final data summaries are provided to the participant as needed.

Compounds received from participants and tested in the ETSP under conditions of confidentiality can come from different points along the drug discovery/development spectrum. Thus, the program is flexible in being able to test compounds that are in early discovery all the way to compounds that are in human clinical trials or even FDA-approved for other indications, and being considered for “repurposing”. A benefit of testing more advanced compounds is that there is a greater body of supporting data (e.g., pharmacokinetics, toxicology, other efficacy measures, etc.) that are available to the NINDS ETSP staff to guide the planning of testing (i.e., dose-selection), thereby improving efficiency, safety, tolerability and interpretability of the test results generated. These supporting data are particularly important as compounds are tested in the newer advanced epilepsy models which can be very resource-intensive, such as the chronic epileptic rat model (see “ETSP Compound Testing Paradigms” section), or more sensitive to pharmacological intervention, such as the kindled rat [3]. The ETSP staff encourages participants to provide such data at appropriate times and is exploring new avenues for generating such information if the participant is not able to readily provide it.

In addition to receiving compounds from external participants, NINDS ETSP staff also proactively evaluate compounds they have independently identified as potential “probes” for exploring novel mechanisms of action. These probe compounds may not be suitable as development candidates, but, if shown to have promising activity in preclinical models, could provide an impetus for external researchers to pursue additional medicinal chemistry efforts to generate more “drug-able” candidates with needed claims for intellectual property. The intent of NINDS is to make these probe compound data available to the public by placing them on the PANAChE website (see “ETSP Compound Testing Paradigms” section below) to encourage further discovery efforts from external investigators.

Based on recommendations made by Landis et al. in 2012 for improving the quality of supported preclinical research through rigorous study design and transparent reporting of data [4], NIH has established best-practice guidelines (https://www.nih.gov/research-training/rigor-reproducibility) directed particularly towards confirmatory (hypothesis testing) experiments [4]. While the scope of the research performed by the ETSP is predominantly exploratory (also referred to as “hypothesis generating”) in nature, the program does strive to implement these guidelines whenever possible. Examples include implementing procedures for quality control on submitted investigational compounds, using blinding procedures, performing dose–response studies, assessing for possible sex differences, and additionally for advanced tests randomizing animals and providing fully powered sample sizes, all with the goal of ultimately reducing the potential for bias and increasing reproducibility. As needed, additional experiments can be designed to generate additional confirmatory data that are fully consistent with NIH guidance.

## ETSP Compound Testing Paradigms

The ETSP currently has efforts aimed at identifying promising agents to address the unmet medical needs in three areas of epilepsy: pharmacoresistant epilepsy, epilepsy prevention (antiepileptogenesis) or modification, and therapies for special patient populations.

### Pharmacoresistant Epilepsy

It is important to emphasize that the Working Groups clearly recognized the important contributions that the ASP had made to providing new symptomatic treatment options to the epilepsy market (see Fig. 3). One recent example is lacosamide, a compound first synthesized by Dr. Harold Kohn and submitted to the ASP for antiseizure activity testing (see commentary in this Special Issue by Dr. Kohn). Many new marketed antiseizure drugs are now available that provide improvements including fewer drug–drug interactions, improved tolerability, and other properties. However, despite the advances made in providing more treatment options, there still remains approximately 30% of people with epilepsy who do not adequately respond to current treatments. Thus, there is a need for new screening strategies and preclinical models to identify compounds that would address this unmet need.

**Fig. 4:**
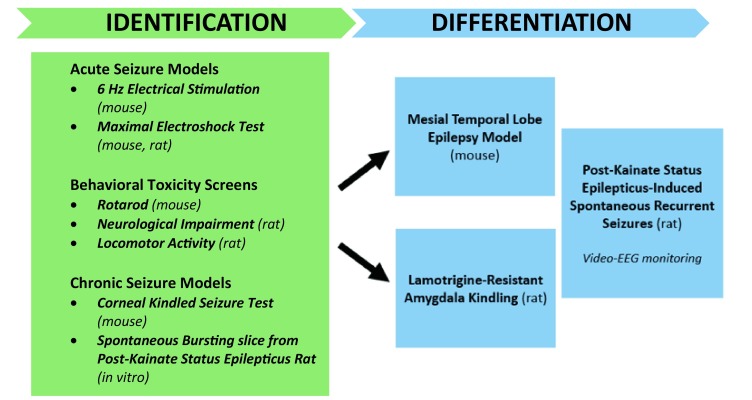
Pharmacoresistance epilepsy work flow for the Epilepsy Therapy Screening Program (ETSP)

In response to this clinical need and based on the input from the Working Groups and the ECB, the ETSP has developed a more refined flow chart (see Fig. 4) to evaluate the potential of new compounds for treating drug refractory epilepsy. Testing is divided into an initial “Identification” phase, followed by a “Differentiation” phase. Evaluation of compounds begins with assessment in two acute seizure models in normal mice, the maximal electroshock (MES) seizure test, and the 6 Hz 44 mA test. The MES test, a measure of generalized tonic-clonic seizures, is sensitive to a wide range of marketed antiseizure agents, whereas the 6 Hz 44 mA test is a model of focal seizures that shows resistance to numerous current antiseizure agents [5]. The inclusion of this “high-hurdle”, acute seizure assay at the initial stage of the Identification phase is intended to raise the threshold for advancing compounds for the management of pharmacoresistant epilepsy, thereby increasing the probability that agents with improved efficacy relative to existing agents will be detected. Good activity in the 6 Hz 44 mA assay can be sufficient to advance a compound into the Differentiation phase.

It is very possible that some compounds with novel mechanisms of action might not be active in the MES or 6 Hz 44 mA tests, but may in fact have potential as improved antiseizure therapeutic agents. For example, such compounds may mechanistically interact with aberrant processes that are present in the epileptic brain, but not in the normal brain of the rodent receiving MES or 6 Hz stimulation. As seen in Fig. 4, there are additional assay options available in the Identification phase to evaluate for activity of such compounds. Of note, these additional assays are models of chronic network hyperexcitability or neuroanatomical remodeling that can occur in the context of recurrent seizures and epilepsy. One of these assays is the corneal kindled mouse, in which the brain has become hyperexcitable and the threshold for electrically-eliciting seizures lowered as the result of repeated corneal electrical stimulation [6]. The other testing platform available is an in vitro assay in which spontaneous electrical bursting occurs in a hippocampal/entorhinal cortex-containing brain slice removed from a rat that has been treated with a regimen of kainic acid that induces status epilepticus [7, 8]. Because these “disease-like” models are also more resource intensive than the acute, electrically-evoked MES or 6 Hz assays, they are used judiciously to evaluate compounds that are not active in the acute seizure screens. The in vitro bursting slice model also has the added advantage of potentially identifying antiseizure activity of compounds that may not readily penetrate the brain following systemic administration and therefore are inactive in the in vivo screens. Altogether, the corneal kindled mouse and spontaneous bursting slice models thus provide an opportunity to potentially discover compounds with disease-specific efficacy, should such compounds fail to otherwise demonstrate activity in the MES and 6 Hz assays conducted in neurologically-intact rodents.

Finally, in addition to antiseizure activity, in vivo assessments of minimal motor impairment are obtained using performance on the rotarod in mice, and locomotor activity and neurological impairment scores in the rat, in addition to overall visual behavioral observations by trained observers. The quantification of key parameters in these assays allows an estimation of the compound’s therapeutic index in either rodent species, e.g., the ratio of the 50% toxic dose (TD_50_) divided by the 50% efficacious dose (ED_50_). Ultimately, the therapeutic index is useful for comparison to clinical antiseizure drugs in the in vivo assays to define the potential for adverse effects.

In summary, the Identification phase of the current pharmacoresistance flow chart has expanded flexibility (relative to previous paradigms) to efficiently identify potentially novel antiseizure agents that can be then advanced to the Differentiation phase. For examples of testing of compounds in this new Identification phase of this flow chart, the reader is referred to manuscripts by Melissa Barker-Haliski et al., and Brian Klein et al. in this special issue.

The Differentiation phase is currently comprised of three assays, one in mice and two in rats. The Differentiation phase models include: the mouse model of mesial Temporal Lobe Epilepsy (mTLE); the lamotrigine-resistant amygdala-kindled rat; and the chronically-epileptic rat. All three models offer the added advantage of being models of chronic seizure activity induced by chemical or electrical insult. Moreover, all three models replicate numerous clinical aspects of epilepsy to provide a more etiologically-relevant approach to the further characterization of promising investigational antiseizure drugs.

In the Differentiation phase assay utilizing a mouse model of mTLE, the efficacy of an investigational compound is measured by its activity in reducing hippocampal spontaneous electrographic seizures (hippocampal paroxysmal discharges, or HPDs) assessed at four weeks following unilateral focal injection of a status-epilepticus inducing dose of kainic acid into the hippocampus [9, 10]. This chronic disease model is considered pharmacoresistant in that some, but not all, antiseizure drugs are effective in reducing HPDs at doses that are not impairing [10]. For additional information concerning clinical TLE and the mTLE mouse model, the reader is referred to the manuscript by Venceslas Duveau and Corinne Roucard in this special issue. The mouse mTLE model was implemented in the ETSP through a competitive subcontract with the French contract research organization, SynapCell (Grenoble, France). Notably, this was the first time a subcontract mechanism had been employed by the program to allow rapid access to a moderate-throughput screening approach not readily available from the prime contractor.

The second model of pharmacoresistant epilepsy used by the ETSP in the Differentiation phase is the lamotrigine-resistant amygdala kindled rat. In this model, rats that are exposed to the antiseizure drug lamotrigine during the process of amygdala kindling subsequently develop a resistance to the antiseizure effects of lamotrigine and other selected sodium-channel blocking drugs [11, 12]. However, these animals retain sensitivity to valproic acid. Thus, the goal of testing in this model is to identify a compound that retains its antiseizure activity in the lamotrigine-resistant rat.

A third disease-relevant model implemented in the Differentiation phase is a well-documented chronic epilepsy rat model. In this model, status epilepticus is induced in rats by systemic administration of kainic acid, and, following a latent period, spontaneous recurrent seizures develop as measured by continuous video/EEG recording. These spontaneous seizures can be highly resistant to some standard antiseizure drugs [13, 14] and therefore, the efficacy of compounds could potentially be measured by their activity in reducing these pharmacoresistant seizures. Moreover, the post-kainic acid status epilepticus rat model of chronic epilepsy exhibits the pathophysiological neuroinflammation consistent with human temporal lobe epilepsy [15], further supporting the utility of this model for drug discovery applications.

Collectively, the data generated from these models create a pharmacological profile that identifies promising investigational compounds for further development and potential treatment of pharmacoresistant epilepsy.

### Special Populations in Epilepsy

A challenge facing new drug therapy discovery in epilepsy is the heterogeneity of the “epilepsies”. For example, according to the recent International League Against Epilepsy (ILAE) commissioned report, there are over 30 epilepsy syndromes [16] and a significant number of genetic mutations associated with epilepsy are being identified [17–19]. Thus, depending upon the underlying causal biology of the disease, treatments for specific syndromes or genetically-based epilepsies may or may not be more widely generalizable to other conditions beyond that specific disorder.

The ETSP has incorporated into its test battery an etiologically-relevant mouse model of epilepsy associated with viral-induced encephalitis. In this model, Theiler’s murine encephalitis virus (TMEV) administered intracranially to C57Bl/6 mice induces transient, acute seizures (4–10 days post injection), which then, after a subsequent latent period, are followed by chronic, recurrent seizures in a subset of animals [20–22]. This model was discovered and characterized at the University of Utah by Dr. Robert Fujinami, Dr. Steve White, and Dr. Karen Wilcox. It is notable that significant neuroinflammation might contribute to transient symptomatic seizures and subsequent epileptogenesis and behavioral deficits [23–25] seen in these mice and therefore, this model may be of particular interest for evaluating compounds that suppress the inflammatory response, which may contribute to epilepsy [24, 26–28]. The ETSP is currently exploring the effects of test compounds on the initial, transient phase of seizure susceptibility [23, 24]. Evaluating compound effects on spontaneous recurrent seizures is more problematic, given their very low rate of occurrence, so additional work is needed to optimize the assay for this purpose.

Finally, the ETSP can also address the pharmacoresistant status epilepticus special population. In the ETSP approach, acute status epilepticus is induced by systemic administration of the chemoconvulsant pilocarpine, and compounds are evaluated for their potential to stop or reduce behavioral seizures. Importantly, testing is performed after pilocarpine administration at a time when the rat has become pharmacoresistant to benzodiazepines such as diazepam or midazolam [29].

### Epilepsy Prevention (Antiepileptogenesis) or Modification

Another area of unmet medical need in epilepsy emphasized by the Working Groups is the demand for treatments for the prevention (antiepileptogenesis) or modification of epilepsy. The ETSP has initiated activity for identifying antiepileptogenic compounds using some of the chronic epilepsy models described above for treatment of pharmacoresistant epilepsy. Two of these models, the mouse mTLE model [10] and the rat chronic epilepsy model, have similarities in that the chronic epileptic disease state is produced as a consequence of initial exposure to status epilepticus-inducing doses of kainic acid, and both models exhibit a latent period before the onset of the spontaneous recurrent electrographic discharges and behavioral seizures, respectively. Thus, a test compound can be administered acutely or chronically at appropriate times after the initial insult and the latency to spontaneous recurrent seizures can be measured. In a similar manner, the potential disease-modifying effect of a test compound can be assessed by beginning dosing at a point when the disease is established, e.g., spontaneous hippocampal paroxysmal discharges (HPDs) or seizure frequency is high. The subsequent effects of the investigational agent on HPDs or seizure expression can then be measured after dosing has been discontinued. Such a drug testing paradigm is thus useful to define the potential activity of a promising investigational agent on established spontaneous network hyperexcitability.

The TMEV mouse model of virus-induced epilepsy is another model which could be potentially used to look at antiepileptogenesis or disease-modifying effects of compounds. Indeed, the ETSP has previously supported such evaluations for the assessment of biomarkers of the epileptogenesis process associated with TLE [23, 24]. However, a challenge associated with the use of this model for true antiepileptogenesis studies (as noted above) is that the frequency of spontaneous recurrent seizures weeks to months after the viral infection period is low [21] and therefore, generating sufficient sample sizes to complete a study would be very resource-intensive.

## PANAChE Database

An important milestone for the ETSP has been the release of a publicly-accessible database referred to by the acronym PANAChE (Public Access to Neuroactive and Anticonvulsant Chemical Evaluations) (http://panache.ninds.nih.gov.).

The PANAChE website is a valuable resource that serves several purposes for the ETSP. First, it provides detailed information on tests, procedures, and work flows used by the ETSP, both currently and historically. Second, it provides a searchable repository for non-confidential efficacy data on compounds tested by the program. This database includes compound structure (and links to the PUBCHEM database) as well as all data (both positive and negative) that have been generated for select compounds in ETSP models performed. The different types of compounds designated for inclusion include: (1) selected standard reference compounds (i.e., antiseizure drugs, or drugs from other therapeutic areas) that have been tested by the ETSP; (2) “probe” compounds (i.e., compounds with a specific mechanism of action) that have been chosen by the ETSP independently of program participants; and (3) test compounds that have been submitted by participants in the program AND for which the submitting participant has provided permission to release the data. With regard to compounds submitted by participants, it is important to emphasize that the ETSP maintains strict confidentiality and will not release information in PANAChE without permission.

The database currently contains data on approximately 200 compounds, but additional compounds are being added over time. It is the intent of NINDS that this database will become increasingly useful as an epilepsy drug discovery tool as more data are released.

## Beyond the ETSP

The ETSP plays an important role as an early drug discovery program, but it is important to emphasize that it is only one step in a larger, more complex system for bringing new antiseizure drugs to the market that may ultimately address unmet medical needs in epilepsy. It is often noted that phenytoin, which was identified in a preclinical MES test in the 1930s [30], advanced to the market in only 1 year. Clearly, the process today is vastly more complex, time-consuming and costly, and the hurdles to the market could be many [31]. Thus, it is important to consider how other programs and resources may complement the data that is generated by the ETSP. As noted earlier, the ETSP is one program that is part of the Division of Translation Research (DTR) at NINDS. Importantly, there are other translational programs that can provide further opportunities for the development of compounds that are tested by the ETSP (https://www.ninds.nih.gov/Current-Research/Research-Funded-NINDS/Translational-Research/Funding-Programs). DTR translational programs generally utilize a milestone-driven approach to progress the most promising projects and de-risk them for further funding and development. Specific funding structures are designed to assist the transition from discovery to development phases with minimal gaps. For small molecules, which comprise the majority of ETSP submissions, the Blueprint Neurotherapeutics Network (BPN) provides funding for late-discovery/early development of small molecules. Entry into this program can occur at multiple stages, but typically occurs at the lead optimization stage. Biologics can be submitted to the Cooperative Research to Enable and Advance Translational Enterprises (CREATE) program tailored for biologics (CREATE-BIO). Although device evaluation is not within the scope of the ETSP, note that there is a separate CREATE program for devices (CREATE-DEVICES). An additional funding opportunity is offered by the Innovation Grants to Nurture Initial Translational Efforts (IGNITE) program, which helps the investigator generate data which will facilitate subsequent application to the advanced translational programs. For small businesses, the Small Business Innovation Research (SBIR) and Small Business Technology Transfer (STTR) programs are potential sources of funding for research and development for products that have the potential for commercialization.

Another program that may be of potential interest to ETSP participants is the NIH Countermeasures Against Chemical Threats (CounterACT) program (https://www.ninds.nih.gov/Current-Research/Trans-Agency-Activities/CounterACT). The overall mission of CounterACT is to develop FDA-approved therapeutics that will reduce mortality and morbidity during and after chemical emergency events. Compounds from ETSP with antiseizure actions may be of interest for evaluation in the CounterACT Neurotherapeutics Screening (CNS) program (https://www.ninds.nih.gov/sites/default/files/CNS_program_0.pdf), which provides initial efficacy screening for compounds that have potential to decrease seizures and neuropathology associated with exposure to organophosphate pesticides and nerve agents. Compounds with appropriate properties have the potential to be developed for eventual inclusion as a standard-of-care therapy to treat the toxic effects of exposure to organophosphate pesticides and nerve agents.

## Final Comments

Significant advances are being made in the understanding of the biology and genetics of the epilepsies, and in generating novel models and workflows for evaluating compounds for their therapeutic potential in treating specific populations. Although the ETSP does not have the resources to incorporate all available models of potential interest and relevance to epilepsy drug discovery, it does have the flexibility to modify its testing schemes to incorporate new models, as consistent with the mission of the program, and is constantly evaluating such possibilities. For example, at present there are no models of pediatric or geriatric epilepsies in the ETSP, but considerations of inclusion of such models can be made. Furthermore, the program has the capability of interacting with other efforts in complementary ways, for example, by alerting ETSP participants to other independent compound testing opportunities. Conversely, compounds emerging from other epilepsy screening efforts that may be targeting specific epilepsy populations not covered by the ETSP could be evaluated in rodent models and paradigms run by the ETSP. Given the diverse number of epilepsy syndromes, exploding knowledge regarding potential genetic etiologies, and inevitable expansion of available animal models, it will be increasingly important to understand the degree to which new agents are specifically effective in models of epilepsy special populations versus their generalizability across broader groups. Coordinating efforts with the many dedicated organizations beyond NIH will be important in generating novel leads and identifying promising agents which will hopefully comprise the next-generation of pharmacological treatments which will significantly reduce the suffering from and burden of epilepsy.
